# Supplementary material for: Serum Long Non-Coding RNAs PVT1, HOTAIR, and NEAT1 as Potential Biomarkers in Egyptian Women with Breast Cancer
Source: Biomolecules. 2021 Feb 18;11(2):301. doi: 10.3390/biom11020301 (PMC7922136; doi:10.3390/biom11020301)
Supplement: Supplementary file 1 [file biomolecules-11-00301-s001.pdf]

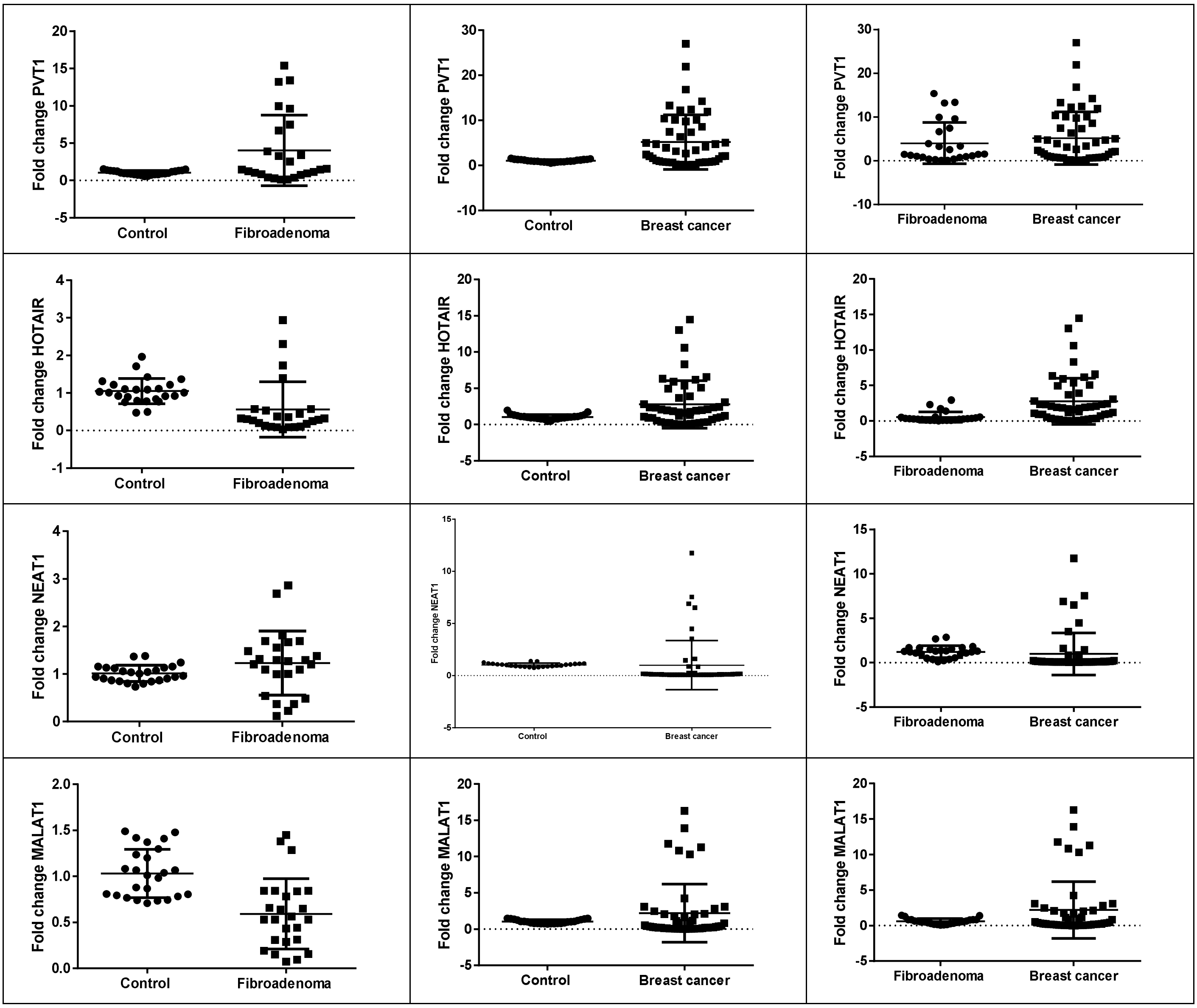

Supplementary Figure 1. Relative expression levels of serum PVT1, HOTAIR, NEAT1, and MALAT1 in the control, fibroadenoma and the breast cancer patients.

Supplementary Table 1. Relative expression levels of serum PVT1, HOTAIR, NEAT1, and MALAT1 in the control, fibroadenoma and the breast cancer patients.

LncRNA	Control	Fibroadenoma	Breast cancer (Age < 50)	*P* value
PVT1	0.9686 (0.8145-1.296)	1.468 [a*] (0.7967-7.092)	4.737 (0.729-9.169)	0.0785
HOTAIR	1.01 (0.815-1.218)	0.31 [a***] (0.116-0.553)	1.202 (0.1452-3.759)	0.0008
NEAT1	1.01 (0.867-1.1360	1.27 [a*] (0.770-1.612)	0.1011[a***,b***] (0.07536-0.1496)	< 0.0001
MALAT1	1.01 (0.783-1.266)	0.53 [a***] (0.299-0.840)	0.2325[a*] (0.1584-1.027)	0.0002

The fold change in gene expression was calculated using $2^{-\Delta\Delta Ct}$ method of Livak and Schmittgen [33] to determine relative quantitative levels of individual lncRNA.

Data are expressed as median (25%-75% percentiles) and were analyzed by Kruskal-Wallis test followed by Dunn's multiple comparisons and Mann-Whitney U tests.

a. Statistical significance from control group

b. Statistical significance from fibroadenoma group

* Significance at $P < 0.05$

*** Significance at $P < 0.0001$.

PVT1, Plasmacytoma variant translocation 1 gene; HOTAIR, HOX transcript antisense RNA; NEAT1, Nuclear enriched abundant transcript 1; MALAT1, Metastasis associated lung adenocarcinoma transcript 1.

Supplementary Table 2. Serum levels of PAI-1 and OPN (ng/ml) in the control, the fibroadenoma and the breast cancer patients.

	Control	Fibroadenoma	Breast cancer	*P* value
**PAI-1**	3.93 ± 3.37	14.38 ± 3.60[a***]	29.84 ± 22.5[a***,b*]	<0.0001
**OPN**	24.91±6.93	27.64±8.702	30.25 ± 6.763	0.1764

Data are expressed as mean ± standard deviation (SD) and were analyzed by ANOVA followed by Sidak’s multiple comparison and Student’s t-tests.

a. Statistical significance from control group

b. Statistical significance from fibroadenoma group

* Significance at $P < 0.05$

*** Significance at $P < 0.0001$

PAI-1, plasminogen activator inhibitor-1; OPN, Osteopontin.
